# How young can children reliably and validly self-report their health-related quality of life?: An analysis of 8,591 children across age subgroups with the PedsQL™ 4.0 Generic Core Scales

**DOI:** 10.1186/1477-7525-5-1

**Published:** 2007-01-03

**Authors:** James W Varni, Christine A Limbers, Tasha M Burwinkle

**Affiliations:** 1 Department of Pediatrics, College of Medicine, Department of Landscape Architecture and Urban Planning, College of Architecture, Texas A&M University, 3137 TAMU, College Station, TX 77843-3137, USA; 2 Department of Psychology, College of Liberal Arts, Texas A&M University, College Station, TX 77843-3137, USA; 3 The Children's Hospital at Scott & White, Department of Pediatrics, College of Medicine, Texas A&M University Health Science Center, 2401 South 31^st ^Street, Temple, TX 76508, USA

## Abstract

**Background:**

The last decade has evidenced a dramatic increase in the development and utilization of pediatric health-related quality of life (HRQOL) measures in an effort to improve pediatric patient health and well-being and determine the value of healthcare services. The emerging paradigm shift toward patient-reported outcomes (PROs) in clinical trials has provided the opportunity to further emphasize the value and essential need for pediatric patient self-reported outcomes measurement. Data from the PedsQL™ Database^SM ^were utilized to test the hypothesis that children as young as 5 years of age can reliably and validly report their HRQOL.

**Methods:**

The sample analyzed represented child self-report age data on 8,591 children ages 5 to 16 years from the PedsQL™ 4.0 Generic Core Scales Database^SM^. Participants were recruited from general pediatric clinics, subspecialty clinics, and hospitals in which children were being seen for well-child checks, mild acute illness, or chronic illness care (n = 2,603, 30.3%), and from a State Children's Health Insurance Program (SCHIP) in California (n = 5,988, 69.7%).

**Results:**

Items on the PedsQL™ 4.0 Generic Core Scales had minimal missing responses for children as young as 5 years old, supporting feasibility. The majority of the child self-report scales across the age subgroups, including for children as young as 5 years, exceeded the minimum internal consistency reliability standard of 0.70 required for group comparisons, while the Total Scale Scores across the age subgroups approached or exceeded the reliability criterion of 0.90 recommended for analyzing individual patient scale scores. Construct validity was demonstrated utilizing the known groups approach. For each PedsQL™ scale and summary score, across age subgroups, including children as young as 5 years, healthy children demonstrated a statistically significant difference in HRQOL (better HRQOL) than children with a known chronic health condition, with most effect sizes in the medium to large effect size range.

**Conclusion:**

The results demonstrate that children as young as the 5 year old age subgroup can reliably and validly self-report their HRQOL when given the opportunity to do so with an age-appropriate instrument. These analyses are consistent with recent FDA guidelines which require instrument development and validation testing for children and adolescents within fairly narrow age groupings and which determine the lower age limit at which children can provide reliable and valid responses across age categories.

## Background

The last decade has evidenced a dramatic increase in the development and utilization of pediatric health-related quality of life measures in an effort to improve pediatric patient health and well-being and to determine the value of healthcare services [1,2]. Health-related quality of life (HRQOL) has been progressively acknowledged as an essential health outcome measure in clinical trials and health services research and evaluation [3-5]. A HRQOL instrument must be multidimensional, consisting at the minimum of the physical, psychological (including emotional and cognitive), and social health dimensions delineated by the World Health Organization [6,7]. Quality of life (QOL) is a broader general conceptual term which encompasses nonhealth-related aspects of life (e.g., the evaluation of the impact of the built environment on general well-being) which are not directly amenable to healthcare services and medical products [7]. Thus, HRQOL has emerged as the most appropriate term for health-related QOL dimensions that represent the patient's perception of the impact of an illness and its treatment and which are thus within the scope of healthcare services and medical products [7].

Although the measurement of HRQOL in pediatric clinical trials has been advocated for a number of years [8], the emerging paradigm shift toward *patient-reported outcomes *(PROs) in clinical trials [7] has provided the opportunity to further emphasize the value and essential need for pediatric patient self-report measurement as efficacy outcomes in clinical trials for pediatric chronic health conditions [9-12].

### Pediatric clinical trials

Historically, only about 20 percent of drugs prescribed for children have been tested for safety and efficacy in pediatric populations and approved by the U.S. Food and Drug Administration (FDA) for labeling claims in pediatric patients [7]. During the past several years, legislative changes have created both voluntary and mandatory guidelines for drug studies in children, resulting in a substantial increase in pediatric clinical trials. Under the Pediatric Exclusivity Provision of the Best Pharmaceuticals for Children Act (BPCA), reauthorized in 2002, companies that conduct drug studies with children, as requested by the FDA, are eligible for an additional six months of marketing exclusivity for the studied drug. The Pediatric Research Equity Act (PREA), signed in 2003, allows the FDA to require pediatric studies if it is determined that the product is likely to be used by a considerable number of pediatric patients, or the product would offer an important advantage to pediatric patients over existing treatments.

While the above pediatric initiatives have created the opportunity for children to be included in clinical trials, pediatric patients have not been afforded the right to self-report on matters pertaining to their health and well-being when evaluating the efficacy of treatments in the vast majority of pediatric clinical trials to date [13]. This fact stands in sharp contrast to the recent FDA draft guidance for industry in which the FDA describes how it evaluates patient-reported outcome (PRO) instruments as efficacy outcomes in clinical trials [7]. In that draft document, the FDA is quite definitive in stating that "some treatment effects are known only to the patient". Thus, what has been an obvious recognition in clinical trials for adult patients, that is, that PROs are *patient *reported outcomes, has not received the same level of recognition in clinical trials for pediatric patients.

### Patient Reported-Outcomes (PROs)

By definition, patient-reported outcomes (PROs) are *self-report *instruments that directly measure the *patient's perceptions *of the impact of disease and treatment as clinical trial endpoints [7]. PROs include multi-item health-related quality of life (HRQOL) instruments, as well as single-item symptom measures (e.g., pain intensity visual analogue scale [VAS]) [14-16]. Research conducted in the 1980's and early 1990's clearly demonstrated that children as young as 5 years of age can self-report their pain intensity using age-appropriate standardized VAS instruments [17-19], establishing pediatric patient self-report of pain intensity as the standard for clinical research and practice. However, young pediatric patients' self-report of their HRQOL at the individual age subgroup level has not been previously reported in the published literature with sufficient sample sizes to support reliability and validity analyses.

### The proxy problem

It is well documented in both the adult and pediatric literature that information provided by proxy-respondents is not equivalent to that reported by the patient [20,21]. Imperfect agreement between self-report and proxy-report, termed cross-informant variance [22], has been consistently documented in the HRQOL measurement of children with chronic health conditions and healthy children [23-30].

In a meta-analysis of studies evaluating the agreement between child self-report and parent proxy-report on different measures of HRQOL, Eiser & Morse (2001) found generally good agreement (*r *> 0.50) between child and parent report for domains reflecting physical activity, functioning and some symptoms, but generally poor agreement (*r *< 0.30) between child self-report and parent proxy-report for emotional and social HRQOL domains [31]. Given these Pearson Product-Moment correlations, and others like them in the literature cited above, it can be concluded that parent proxy-reports typically explain only 10–25% of the variance in child self-report HRQOL outcomes. Thus, the findings on the *proxy problem *"indicate that parent reports cannot be substituted for child reports" [32]. To further complicate the use of proxy reporters, which typically are the child's parents, most often mothers, are the unresolved concerns regarding the influence of parental distress and related factors on parents' perceptions of child health and well-being [33-35].

Taken together, the evidence is quite compelling that evaluating pediatric patients' perspectives regarding treatment efficacy should become the standard in pediatric clinical trials given the potential for a significant degree of measurement error associated with parent proxy-report of child HRQOL. At the very least, parent proxy-report should be included to complement pediatric patient self-report as a secondary outcome measure, not to serve as a convenient substitute or proxy for pediatric patient PROs in pediatric clinical trials. Parent proxy-report should only be the primary outcome measure when the child is too young or ill or otherwise unable to self-report [36].

Recent FDA guidelines recommend that instrument development and validation testing for children and adolescents be conducted within fairly narrow age groupings and to determine the lower age limit at which children can provide reliable and valid responses that can be compared across age categories [7]. Consistent with these recommendations, it has been an explicit goal of the PedsQL™ Measurement Model [24] to develop and test brief measures for the broadest age group empirically feasible, specifically including pediatric patient self-report for the youngest children possible. This goal was originally articulated in the empirical efforts of the 1980's to measure pain perception in pediatric patients through the development and testing of the Varni/Thompson Pediatric Pain Questionnaire™, which included pain intensity visual analogue scales for children as young as 5 years of age [18].

The PedsQL™ 4.0 Generic Core Scales include child self-report for ages 5–18 and parent proxy-report for ages 2–18 [37,38]. The items chosen for inclusion were initially derived from the measurement properties of the child self-report scales, while the parent proxy-report scales were constructed to directly parallel the child self-report items. Thus, the development and testing of the PedsQL™ as a pediatric PRO explicitly emphasizes the child's perceptions, including children as young as 5 years of age, and consequently is an ideal HRQOL instrument to test the lower age limits achievable for pediatric patient HRQOL self-report.

Therefore, the objectives of the current analyses are to determine the feasibility, reliability and validity of child self-report at the individual age subgroup level for children 5–16 years of age utilizing data from the PedsQL™ 4.0 Generic Core Scales Database^SM ^on over 8,500 children and adolescents. These analyses are consistent with the FDA guidelines recommending validation testing for children and adolescents within fairly narrow age groupings and the determination of the lower age limit at which children can provide reliable and valid responses [7].

## Method

### Participants and settings

The sample contains composite child self-report and parent proxy-report age subgroup data on 8,591 children ages 5 to 16 years from the PedsQL™ 4.0 Generic Core Scales Database^SM ^(previously published data, n = 8,086, 94.1%; unpublished data, n = 505, 5.9%). Participants were recruited from general pediatric clinics, subspecialty clinics, and hospitals in which children were being seen for well-child checks, mild acute illness, or chronic illness care (n = 2,603, 30.3%), and from a State Children's Health Insurance Program (SCHIP) in California (n = 5,988, 69.7%). Participants recruited from general pediatric clinics, subspecialty clinics, and hospitals were assessed in-person or by telephone. For in-person mode of administration, research assistants obtained written parental informed consent and child assent. Paper-and-pencil questionnaires were self-administered for parents and for children ages 8 to 16 and interview administered for children ages 5 to 7 and in situations in which the child was unable to read or write as a consequence of either physical or cognitive impairment. For telephone administration, parents of children ages 5 to 16 were called by a research assistant who explained the study, and obtained verbal parental informed consent and child assent. The research assistant verbally administered the PedsQL™ 4.0 individually to the parent and their child. If the child was not home at the time of the initial call, the research assistant arranged for a call at another time. These research protocols were approved by the Institutional Review Board at Children's Hospital and Health Center, San Diego and other appropriate local Institutional Review Boards.

Participants recruited from the State Children's Health Insurance Program (SCHIP) were assessed via statewide mailing. PedsQL™ 4.0 paper-and-pencil surveys were mailed separately for each of the months of February and March 2001 to families with children ages 5–16 years throughout the State of California who were all new enrollees in SCHIP. Parents and children ages 8–16 were instructed to complete the survey separately, while parents of children ages 5–7 were instructed to assist their child in completing the questionnaire after completing the proxy-report. A reminder postcard followed the initial mailing, with a second survey mailed to nonrespondents. Nonrespondents to the second survey received a telephone reminder. Given that this project was conducted for program evaluation to comply with California Insurance Code 12693.92 (b), and not specifically research purposes, parents and children did not complete informed consent forms [38]. This protocol of analyzing existing deidentified data was approved by the Institutional Review Board at Children's Hospital and Health Center, San Diego.

For all forms combined (N = 8,591), the number of children within each age subgroup is as follows: 757 five-year-olds (8.8%), 932 six-year-olds (10.8%), 891 seven-year-olds (10.4%), 882 eight-year-olds (10.3%), 841 nine-year-olds (9.8%), 841 ten-year-olds (9.8%), 683 eleven-year-olds (7.9%), 683 twelve-year-olds (7.9%), 614 thirteen-year-olds (7.1%), 572 fourteen-year-olds (6.7%), 563 fifteen-year-olds (6.6%), and 332 sixteen-year-olds (3.9%). The sample contains 4,391 boys (51.1%), 4,185 girls (48.7%), and 15 missing (0.2%). The sample is heterogeneous with respect to race/ethnicity with 4,403 Hispanics (51.3%), 1,995 White non-Hispanics (23.2%), 759 Asian or Pacific Islanders (8.8%), 405 Black non-Hispanics (4.7%), 41 American Indians or Alaskan Natives (0.5%), 115 other (1.3%), and 873 missing (10.2%). Child surveys were completed in English (n = 4,859, 56.6%), Spanish (n = 3,377, 39.3%), Chinese (n = 184, 2.1%), Korean (n = 93, 1.1%), and Vietnamese (n = 46, 0.5%; missing = 32, 0.4%). Response equivalence has been previously demonstrated across language for the PedsQL™ by examining the percent missing data, floor and ceiling effects, and scale internal consistency across language, as well as across mode of administration [37].

The sample included healthy children, who were assessed either in physicians' offices during well-child checks and/or whose parents did not report the presence of a chronic health condition (n = 5,491, 63.9%), acutely ill children, whose parents did not report the presence of a chronic health condition, but who were assessed at one of the pediatric clinics or hospitals (n = 142, 1.7%), chronically ill children, whose parents reported the presence of a chronic health condition (i.e., a physical or mental health condition that has lasted or is expected to last at least 6 months and interferes with the child's activities) and/or were identified through their medical records as having a chronic health condition (n = 2,627, 30.6%), and 331 missing (3.9%). Within each age subgroup, the number of healthy and chronically ill children is as follows: 561 healthy (74.1%) and 155 chronically ill (20.5%) five-year-olds, 717 healthy (76.9%) and 161 chronically ill (17.3%) six-year-olds, 646 healthy (72.5%) and 185 chronically ill (20.8%) seven-year-olds, 590 healthy (66.9%) and 252 chronically ill (28.6%) eight-year-olds, 558 healthy (66.3%) and 233 chronically ill (27.7%) nine-year-olds, 545 healthy (64.8%) and 256 chronically ill (30.4%) ten-year-olds, 404 healthy (59.2%) and 257 chronically ill (37.6%) eleven-year-olds, 415 healthy (60.8%) and 225 chronically ill (32.9%) twelve-year-olds, 326 healthy (53.1%) and 258 chronically ill (42.0%) thirteen-year-olds, 301 healthy (52.6%) and 238 chronically ill (41.6%) fourteen-year-olds, 289 healthy (51.3%) and 230 chronically ill (40.9%) fifteen-year-olds, and 139 healthy (41.9%) and 177 chronically ill (53.3%) sixteen-year-olds. The chronically ill sample (n = 2,627) is heterogeneous in terms of diagnoses with 374 children diagnosed with asthma (14.2%), 358 with cancer (13.6%), 291 with diabetes (11.1%), 269 with a gastrointestinal condition (10.2%), 268 with a rheumatic condition (10.2%), 199 with a cardiac condition (7.6%), 103 diagnosed as obese (3.9%), 103 with sickle cell anemia (3.9%), 78 with ADHD (3.0%), 72 with renal disease (2.7%), 71 with cerebral palsy (2.7%), 45 with mental health conditions (1.7%), and 396 with other chronic conditions (15.1%).

## Measures

### The PedsQL™ 4.0 (Pediatric Quality of Life Inventory™ Version 4.0)

The 23-item PedsQL™ 4.0 Generic Core Scales encompass: 1) Physical Functioning (8 items), 2) Emotional Functioning (5 items), 3) Social Functioning (5 items), and 4) School Functioning (5 items), and were developed through focus groups, cognitive interviews, pre-testing, and field testing measurement development protocols [24,37]. The instrument takes approximately 5 minutes to complete [37].

The PedsQL™ 4.0 Generic Core Scales are comprised of parallel child self-report and parent proxy-report formats. Child self-report includes ages 5–7, 8–12, and 13–18 years. Parent proxy-report includes ages 2–4 (toddler), 5–7 (young child), 8–12 (child), and 13–18 (adolescent), and assesses parent's perceptions of their child's HRQOL. The items for each of the forms are essentially identical, differing in developmentally appropriate language, or first or third person tense. The instructions ask how much of a problem each item has been during the past one month. A 5-point Likert response scale is utilized across child self-report for ages 8–18 and parent proxy-report (0 = never a problem; 1 = almost never a problem; 2 = sometimes a problem; 3 = often a problem; 4 = almost always a problem). To further increase the ease of use for the young child self-report (ages 5–7), the response scale is reworded and simplified to a 3-point scale (0 = not at all a problem; 2 = sometimes a problem; 4 = a lot of a problem), with each response choice anchored to a happy to sad faces scale [18,39]. For the purposes of these analyses, parent proxy-report data for ages 5–16 were matched to the child self-report data.

Items are reverse-scored and linearly transformed to a 0–100 scale (0 = 100, 1 = 75, 2 = 50, 3 = 25, 4 = 0), so that higher scores indicate better HRQOL. Scale Scores are computed as the sum of the items divided by the number of items answered (this accounts for missing data). If more than 50% of the items in the scale are missing, the Scale Score is not computed. This accounts for the differences in sample sizes for scales reported in the Tables. Although there are other strategies for imputing missing values, this computation is consistent with the previous PedsQL™ peer-reviewed publications, as well as other well-established HRQOL measures [37,40,41]. For this study, over 99% of child respondents were included in the Scale Score analyses after imputing missing values. The Physical Health Summary Score (8 items) is the same as the Physical Functioning Scale. To create the Psychosocial Health Summary Score (15 items), the mean is computed as the sum of the items divided by the number of items answered in the Emotional, Social, and School Functioning Scales.

### PedsQL™ Family Information Form

The PedsQL™ Family Information Form [37] or survey items adapted from the PedsQL™ Family Information Form were completed by parents. The PedsQL™ Family Information Form contains demographic information including the child's date of birth, gender, race/ethnicity, and parental education and occupation information required to calculate the Hollingshead socioeconomic status (SES) index [42]. One survey question asks the parent to report on the presence of a chronic health condition ("In the past 6 months, has your child had a chronic health condition?") defined as a physical or mental health condition that has lasted or is expected to last at least 6 months and interferes with the child's activities. If the parents check "Yes" to this question, they are asked to write in the name of the chronic health condition.

### Statistical analyses

The feasibility of child self-report was determined from the percentage of missing values for the child self-report sample as a whole and across each individual age subgroup from 5 to 16 years [40]. Scale internal consistency reliability was determined by calculating Cronbach's coefficient alpha across individual age subgroups [43]. Scales with reliabilities of 0.70 or greater are recommended for comparing patient groups, while a reliability criterion of 0.90 is recommended for analyzing individual patient scale scores [44,45]. Range of measurement was based on the percentage of scores at the extremes of the scaling range, that is, the maximum possible score (ceiling effect) and the minimum possible score (floor effect) [40].

Construct validity was determined utilizing the known-groups method. The known-groups method compares scale scores across groups known to differ in the health construct being investigated. In this study, PedsQL™ 4.0 Generic Core Scales Scores in groups differing in known health condition (healthy children and children known to have a chronic illness) were computed across each age subgroup [40,46], using independent sample *t*-tests. In order to determine the magnitude of the anticipated differences, effect sizes were calculated [47]. Effect size as used in these analyses was calculated by taking the difference between the healthy sample mean and the chronic sample mean, divided by the healthy sample standard deviation. Effect sizes for differences in means are designated as small (0.20), medium (0.50), and large (0.80) in magnitude [47].

Agreement between child self-report and parent proxy-report was determined through two-way mixed effect model (absolute agreement, single measure) Intraclass Correlations (ICC) [48]. The ICC offers an index of absolute agreement given that it takes into account the ratio between subject variability and total variability [48,49]. Intraclass Correlations (ICC) are designated as ≤ 0.40 poor to fair agreement, 0.41–0.60 moderate agreement, 0.61–0.80 good agreement, and 0.81–1.00 excellent agreement [50,51]. Statistical analyses were conducted using SPSS Version 13.0 for Windows.

## Results

### Feasibility

The percentage of missing item responses for the child self-report sample as a whole was 1.2%. Items on the PedsQL™ 4.0 Generic Core Scales had minimal missing responses for children as young as 5 years old, with the percentage of missing item responses decreasing slightly with age. The percentage of missing item responses across the age subgroups was 2.8%, 1.3%, 1.5%, 1.5%, 1.0%, 0.99%, 0.85%, 0.95%, 0.76%, 0.95%, 0.95%, and 0.79% for age subgroups 5, 6, 7, 8, 9, 10, 11, 12, 13, 14, 15, and 16, respectively. It should be noted that most of the missing data for ages 5–7 involved the School Functioning Scale (60.0%, 26.6%, and 34.5% for ages 5, 6, and 7, respectively). This is not a surprising finding, since young children do not necessarily attend school. When eliminating the School Functioning items, the percentage of missing items for the Total Scale Score is 1.1%, 0.98%, and 0.99% for ages 5, 6, and 7, respectively.

### Internal consistency reliability

Internal consistency reliability alpha coefficients across individual age subgroups are presented for the PedsQL™ 4.0 Generic Core Scales Total Scale Score in Table 1, Physical Health Summary Score in Table 2, Psychosocial Health Summary Score in Table 3, Emotional Functioning Scale Score in Table 4, Social Functioning Scale Score in Table 5, and School Functioning Scale Score in Table 6. The majority of the child self-report scales across the age subgroups, including for children as young as 5 years, exceeds the minimum reliability standard of 0.70 required for group comparisons, while the Total Scale Scores across the age subgroups approaches or exceeds the reliability criterion of 0.90 recommended for analyzing individual patient scale scores. Alpha values are lower for the School Functioning Scale Scores across the age subgroups. The alpha value is lowest on the School Functioning Scale for the 6 year old subgroup. Across the PedsQL™ scales and summary scores, internal consistency reliability alpha coefficients increase slightly with age.

**Table 1:** PedsQL™ 4.0 Generic Core Scales Total Scale Score: Child Self-Report Reliability and Validity

**Age**	**Reliability**		**Validity**									
			**Chronic Health Condition**				**Healthy Sample**					

	n	α	Mean	SD	% Floor	% Ceiling	Mean	SD	% Floor	% Ceiling	Chronic vs. Healthy Difference	Chronic vs. Healthy Effect Size

5 yrs	693	0.86	74.83	15.41	0.0	2.6	83.22	12.18	0.0	8.2	8.39*	0.69
6 yrs	913	0.86	76.24	14.82	0.0	4.3	82.12	12.73	0.0	7.1	5.88*	0.46
7 yrs	869	0.87	71.21	15.42	0.0	0.0	80.98	12.98	0.0	7.4	9.77*	0.75
8 yrs	864	0.90	75.43	14.73	0.0	1.6	83.54	12.95	0.0	8.6	8.11*	0.63
9 yrs	827	0.91	74.16	15.75	0.0	1.7	83.71	13.76	0.0	7.2	9.55*	0.69
10 yrs	825	0.90	76.13	15.84	0.0	3.1	84.16	12.72	0.0	9.5	8.03*	0.63
11 yrs	675	0.91	76.63	15.56	0.0	2.0	85.61	12.47	0.0	9.7	8.98*	0.72
12 yrs	669	0.91	76.57	15.48	0.0	1.3	84.01	12.97	0.0	9.9	7.44*	0.57
13 yrs	609	0.92	75.08	16.06	0.0	3.1	84.23	13.15	0.0	9.8	9.15*	0.70
14 yrs	560	0.92	74.10	16.34	0.0	2.9	85.71	11.97	0.0	11.6	11.61*	0.97
15 yrs	554	0.91	75.03	14.97	0.0	0.9	84.70	12.73	0.0	12.8	9.68*	0.76
16 yrs	327	0.91	74.67	16.52	0.0	0.6	85.76	11.41	0.0	10.1	11.09*	0.97

Note: Total N = 8,385 for reliability, Total N = 8,098 for validity.

Higher values equal better health-related quality of life.

% Floor/Ceiling = the percentage of scores at the extremes of the scaling range.

Effect sizes are designated as small (.20), medium (.50), and large (.80).

*p < .001 (independent samples t-test).

**Table 2:** PedsQL™ 4.0 Generic Core Scales Physical Health Summary Score: Child Self-Report Reliability and Validity

**Age**	**Reliability**		**Validity**									
			**Chronic Health Condition**				**Healthy Sample**					

	n	α	Mean	SD	% Floor	% Ceiling	Mean	SD	% Floor	% Ceiling	Chronic vs. Healthy Difference	Chronic vs. Healthy Effect Size

5 yrs	753	0.72	77.65	19.42	0.0	11.6	86.34	13.38	0.0	25.7	8.69*	0.65
6 yrs	928	0.70	80.31	17.20	0.0	16.7	86.18	13.23	0.0	23.8	5.87*	0.44
7 yrs	885	0.73	75.28	19.18	0.0	8.1	85.52	13.73	0.0	26.0	10.24*	0.75
8 yrs	875	0.79	77.47	17.73	0.0	10.7	87.36	13.77	0.0	28.8	9.88*	0.72
9 yrs	838	0.83	76.37	18.45	0.0	10.7	88.43	14.80	0.0	32.4	12.06*	0.81
10 yrs	837	0.82	77.47	19.34	0.0	13.6	88.03	13.37	0.0	30.6	10.56*	0.79
11 yrs	681	0.84	78.34	19.29	0.0	10.5	88.03	13.30	0.0	29.0	9.68*	0.73
12 yrs	679	0.83	77.92	18.84	0.0	12.4	87.13	13.95	0.0	26.7	9.21*	0.66
13 yrs	614	0.84	76.16	19.19	0.0	11.6	87.83	13.57	0.0	34.0	11.67*	0.86
14 yrs	570	0.87	74.37	22.05	0.0	11.3	89.00	13.20	0.0	30.6	14.62*	1.11
15 yrs	560	0.85	77.21	18.58	0.0	10.9	88.59	13.55	0.0	34.9	11.38*	0.84
16 yrs	331	0.87	75.55	21.75	0.0	12.4	89.13	12.74	0.0	30.2	13.58*	1.07

Note: Total N = 8,551 for reliability, Total N = 8,325 for validity.

Higher values equal better health-related quality of life.

% Floor/Ceiling = the percentage of scores at the extremes of the scaling range.

Effect sizes are designated as small (.20), medium (.50), and large (.80).

*p < .001 (independent samples t-test).

**Table 3:** PedsQL™ 4.0 Generic Core Scales Psychosocial Health Summary Score: Child Self-Report Reliability and Validity

**Age**	**Reliability**		**Validity**									
			**Chronic Health Condition**				**Healthy Sample**					

	n	α	Mean	SD	% Floor	% Ceiling	Mean	SD	% Floor	% Ceiling	Chronic vs. Healthy Difference	Chronic vs. Healthy Effect Size

5 yrs	696	0.82	73.16	16.30	0.0	3.9	81.56	13.76	0.0	11.6	8.40*	0.61
6 yrs	914	0.82	74.06	16.13	0.0	5.6	79.91	14.40	0.0	9.8	5.85*	0.41
7 yrs	870	0.83	69.14	16.68	0.0	0.0	78.55	14.73	0.0	8.7	9.41*	0.64
8 yrs	867	0.86	74.27	15.53	0.0	2.4	81.53	14.25	0.0	10.3	7.26*	0.51
9 yrs	829	0.87	73.01	16.90	0.0	2.6	81.20	14.99	0.0	8.8	8.19*	0.55
10 yrs	829	0.87	75.44	16.82	0.0	3.5	82.09	14.06	0.0	10.5	6.65*	0.47
11 yrs	675	0.89	75.77	16.41	0.0	2.8	84.32	13.45	0.0	11.1	8.55*	0.64
12 yrs	672	0.88	75.86	16.14	0.0	1.8	82.20	14.45	0.0	13.0	6.33*	0.44
13 yrs	610	0.89	74.46	16.69	0.0	5.4	82.34	14.69	0.0	12.0	7.87*	0.54
14 yrs	562	0.88	73.99	16.20	0.0	5.4	83.98	13.28	0.0	12.6	9.99*	0.75
15 yrs	556	0.88	73.89	15.31	0.0	1.3	82.72	14.21	0.0	14.9	8.83*	0.62
16 yrs	328	0.88	74.21	16.36	0.0	1.7	83.98	13.03	0.0	10.8	9.78*	0.75

Note: Total N = 8,408 for reliability, Total N = 8,324 for validity.

Higher values equal better health-related quality of life.

% Floor/Ceiling = the percentage of scores at the extremes of the scaling range.

Effect sizes are designated as small (.20), medium (.50), and large (.80).

*p < .001 (independent samples t-test).

**Table 4:** PedsQL™ 4.0 Generic Core Scales Emotional Functioning Scale Score: Child Self-Report Reliability and Validity

**Age**	**Reliability**		**Validity**									
			**Chronic Health Condition**				**Healthy Sample**					

	n	α	Mean	SD	% Floor	% Ceiling	Mean	SD	% Floor	% Ceiling	Chronic vs. Healthy Difference	Chronic vs. Healthy Effect Size

5 yrs	750	0.70	72.83	23.36	0.0	20.6	79.11	17.88	0.2	24.4	6.28**	0.35
6 yrs	926	0.70	75.45	21.73	0.0	24.1	78.73	18.46	0.1	24.7	3.29*	0.18
7 yrs	886	0.73	69.44	22.10	1.1	13.4	77.38	19.07	0.0	24.6	7.95**	0.42
8 yrs	874	0.75	70.96	19.37	0.0	10.3	77.53	18.97	0.2	20.8	6.57**	0.35
9 yrs	840	0.77	72.01	21.17	0.0	14.1	78.17	18.28	0.2	19.7	6.16**	0.34
10 yrs	840	0.77	73.93	19.94	0.0	14.8	79.88	17.72	0.0	22.0	5.96**	0.34
11 yrs	680	0.79	74.60	20.41	0.4	15.3	81.04	17.51	0.0	25.2	6.44**	0.37
12 yrs	678	0.79	73.68	19.72	0.0	12.8	79.22	18.24	0.2	21.7	5.54**	0.30
13 yrs	615	0.81	72.55	21.22	0.0	13.5	80.13	18.15	0.0	23.9	7.57**	0.42
14 yrs	572	0.79	72.92	20.22	0.4	14.6	81.02	17.78	0.3	23.6	8.10**	0.46
15 yrs	562	0.81	70.90	21.20	0.9	13.5	79.72	18.57	0.0	27.3	8.82**	0.47
16 yrs	332	0.81	70.74	21.97	0.6	14.0	80.15	17.99	0.0	20.9	9.41**	0.52

Note: Total N = 8,555 for reliability, Total N = 8,319 for validity.

Higher values equal better health-related quality of life.

% Floor/Ceiling = the percentage of scores at the extremes of the scaling range.

Effect sizes are designated as small (.20), medium (.50), and large (.80).

*p < .05, **p < .001 (independent samples t-test).

**Table 5:** PedsQL™ 4.0 Generic Core Scales Social Functioning Scale Score: Child Self-Report Reliability and Validity

**Age**	**Reliability**		**Validity**									
			**Chronic Health Condition**				**Healthy Sample**					

	n	α	Mean	SD	% Floor	% Ceiling	Mean	SD	% Floor	% Ceiling	Chronic vs. Healthy Difference	Chronic vs. Healthy Effect Size

5 yrs	749	0.68	74.13	19.31	0.6	15.5	83.39	16.82	0.0	34.8	9.26*	0.55
6 yrs	925	0.68	75.87	20.29	0.0	22.2	81.06	17.73	0.1	29.8	5.19*	0.29
7 yrs	885	0.71	69.58	22.10	1.6	13.4	80.25	18.02	0.0	27.7	10.67*	0.59
8 yrs	876	0.74	78.72	18.65	0.4	19.4	85.00	16.58	0.0	34.1	6.28*	0.38
9 yrs	837	0.79	77.21	20.73	0.0	20.1	84.16	18.54	0.2	36.0	6.96*	0.38
10 yrs	837	0.77	80.08	20.36	0.4	26.1	85.47	16.84	0.0	35.4	5.39*	0.32
11 yrs	680	0.80	80.47	19.65	0.0	25.4	88.53	14.99	0.0	43.6	8.06*	0.54
12 yrs	679	0.78	81.78	19.75	0.0	27.9	87.27	15.47	0.0	37.3	5.48*	0.35
13 yrs	612	0.81	81.32	18.25	0.0	25.9	86.84	16.47	0.0	40.8	5.52*	0.34
14 yrs	571	0.83	78.88	21.53	0.8	25.9	89.82	14.65	0.0	49.2	10.94*	0.75
15 yrs	562	0.81	82.99	17.59	0.0	30.0	89.06	14.49	0.0	45.7	6.07*	0.42
16 yrs	331	0.77	83.37	17.69	0.0	27.0	90.29	12.63	0.0	46.8	6.92*	0.55

Note: Total N = 8,544 for reliability, Total N = 8,308 for validity.

Higher values equal better health-related quality of life.

% Floor/Ceiling = the percentage of scores at the extremes of the scaling range.

Effect sizes are designated as small (.20), medium (.50), and large (.80).

*p < .001 (independent samples t-test).

**Table 6:** PedsQL™ 4.0 Generic Core Scales School Functioning Scale Score: Child Self-Report Reliability and Validity

**Age**	**Reliability**		**Validity**									
			**Chronic Health Condition**				**Healthy Sample**					

	n	α	Mean	SD	% Floor	% Ceiling	Mean	SD	% Floor	% Ceiling	Chronic vs. Healthy Difference	Chronic vs. Healthy Effect Size

5 yrs	702	0.63	72.24	20.78	0.0	13.5	82.02	15.92	0.0	22.5	9.77*	0.61
6 yrs	920	0.59	71.81	18.11	0.0	10.5	79.93	16.56	0.1	21.6	8.12*	0.49
7 yrs	873	0.62	68.15	18.15	0.0	5.4	77.90	17.16	0.0	18.4	9.76*	0.57
8 yrs	872	0.68	73.27	17.39	0.0	6.7	82.11	15.56	0.0	21.0	8.84*	0.57
9 yrs	831	0.74	69.75	19.59	0.0	5.1	81.05	17.15	0.4	18.1	11.30*	0.66
10 yrs	832	0.72	72.76	19.45	0.4	8.9	80.95	16.32	0.0	19.4	8.19*	0.50
11 yrs	676	0.76	72.37	19.37	0.4	7.7	83.43	15.56	0.0	23.8	11.05*	0.71
12 yrs	677	0.75	71.94	19.30	0.4	8.4	80.25	17.26	0.0	20.5	8.30*	0.48
13 yrs	612	0.79	69.56	21.34	0.0	8.9	80.17	17.13	0.0	19.3	10.61*	0.62
14 yrs	563	0.76	69.94	18.14	0.0	8.4	81.17	17.07	0.3	21.9	11.23*	0.66
15 yrs	556	0.78	67.78	19.02	0.0	5.2	79.33	17.85	0.0	20.8	11.55*	0.65
16 yrs	328	0.77	68.36	19.93	0.6	5.6	81.49	17.41	0.0	23.0	13.12*	0.75

Note: Total N = 8,442 for reliability, Total N = 8,211 for validity.

Higher values equal better health-related quality of life.

% Floor/Ceiling = the percentage of scores at the extremes of the scaling range.

Effect sizes are designated as small (.20), medium (.50), and large (.80).

*p < .001 (independent samples t-test).

### Range of measurement

Tables 1 through 6 present the percentages of scores at the floor and ceiling for healthy children and children with a chronic health condition across the age subgroups. There were no significant floor effects for healthy children or children with a chronic health condition across the age subgroups, with the majority of scales demonstrating 0.0% of respondents scoring at the minimum. Ceiling effects existed in some scales. These ranged from minimal (e.g., 7.1% of healthy respondents in the 6 year old subgroup for the self-report Total Scale Score) to moderate (e.g., 49.2% of healthy respondents in the 14 year old subgroup for the self-report Social Functioning Scale). The ceiling effects were in the expected direction, with healthy children reporting more ceiling effects than children with a chronic health condition. Across the scales and summary scores, for both healthy children and children with a chronic health condition, the percentage of scores at the floor and ceiling was not greater for children in the younger age groups, suggesting that children of younger ages were not navigating to the ends of the response scales, reducing them to essentially yes/no answers. Thus, even the 3-point Likert response scale for ages 5–7 produced a range of responses.

### Construct validity

Tables 1 through 6 demonstrate comparisons between children's self-reported PedsQL™ 4.0 Generic Core Scales Total Scale Scores, Physical Health Summary Scores, Psychosocial Health Summary Scores, Emotional Functioning Scale Scores, Social Functioning Scale Scores, and School Functioning Scale Scores for healthy children and children with a known chronic health condition by individual age subgroups. For each PedsQL™ scale and summary score, across each age subgroup, including children as young as 5 years, healthy children demonstrated a statistically significant difference in HRQOL (better HRQOL) than children with a known chronic health condition, with most effect sizes in the medium to large effect size range [47].

### Parent/child agreement

Table 7 shows two-way mixed effect model (absolute agreement, single measure) Intraclass Correlations (ICC) between PedsQL™ 4.0 child self-report and parent proxy-report across individual age subgroups for the entire sample. Most ICCs are in the range of moderate to good agreement, with ICC's generally increasing with the child's age across the scales and summary scores.

**Table 7:** Two-way mixed effect model (absolute agreement, single measure) Intraclass Correlations (ICC) between Child Self-Report and Parent Proxy-Report for PedsQL™ 4.0 Generic Core Scales by Age for Entire Sample

**Age**	**n**	**Total Score**	**Physical Health**	**Psychosocial Health**	**Emotional Functioning**	**Social Functioning**	**School Functioning**
5 yrs	748	0.51*	0.36*	0.56*	0.62*	0.43*	0.45*
6 yrs	912	0.44*	0.28*	0.50*	0.60*	0.38*	0.44*
7 yrs	873	0.46*	0.31*	0.53*	0.59*	0.41*	0.42*
8 yrs	863	0.57*	0.46*	0.60*	0.63*	0.50*	0.48*
9 yrs	830	0.60*	0.48*	0.63*	0.63*	0.53*	0.57*
10 yrs	826	0.70*	0.60*	0.71*	0.69*	0.63*	0.63*
11 yrs	671	0.62*	0.52*	0.64*	0.63*	0.55*	0.59*
12 yrs	660	0.67*	0.57*	0.69*	0.67*	0.59*	0.64*
13 yrs	605	0.67*	0.57*	0.69*	0.68*	0.57*	0.60*
14 yrs	554	0.70*	0.63*	0.70*	0.67*	0.62*	0.64*
15 yrs	543	0.70*	0.60*	0.71*	0.69*	0.60*	0.66*
16 yrs	321	0.69*	0.62*	0.67*	0.64*	0.53*	0.66*

Note: N = 8,406.

Intraclass Correlation Coefficients (ICC) are designated as ≤ 0.40 poor to fair agreement, 0.41–0.60 moderate agreement, 0.61–0.80 good agreement, and 0.81–1.00 excellent agreement.

*p < .001.

To investigate the relationship between the child's health status and parent-child agreement, Table 8 presents two-way mixed effect model (absolute agreement, single measure) Intraclass Correlations (ICC) between PedsQL™ 4.0 child self-report and parent proxy-report across individual age subgroups for the chronic health condition sample, while Table 9 presents the ICCs for the healthy sample. For both the chronic health condition and healthy samples, the ICCs generally increase with the child's age across the scales and summary scores. Across the age subgroups, the ICCs are generally greater in the chronic health condition sample compared to the healthy sample for the Physical Health Summary Score. ICCs across the age subgroups are generally greater in the healthy sample compared to the chronic health condition sample for the Emotional Functioning Scale.

**Table 8:** Two-way mixed effect model (absolute agreement, single measure) Intraclass Correlations (ICC) between Child Self-Report and Parent Proxy-Report for PedsQL™ 4.0 Generic Core Scales by Age for Chronic Health Condition Sample

**Age**	**n**	**Total Score**	**Physical Health**	**Psychosocial Health**	**Emotional Functioning**	**Social Functioning**	**School Functioning**
5 yrs	151	0.50*	0.46*	0.47*	0.48*	0.35*	0.32*
6 yrs	159	0.37*	0.34*	0.38*	0.43*	0.30*	0.41*
7 yrs	181	0.51*	0.50*	0.48*	0.37*	0.46*	0.33*
8 yrs	251	0.55*	0.52*	0.51*	0.45*	0.45*	0.44*
9 yrs	229	0.54*	0.55*	0.49*	0.45*	0.41*	0.48*
10 yrs	254	0.64*	0.65*	0.60*	0.49*	0.57*	0.59*
11 yrs	241	0.63*	0.64*	0.58*	0.50*	0.57*	0.56*
12 yrs	214	0.61*	0.60*	0.59*	0.54*	0.56*	0.58*
13 yrs	255	0.68*	0.63*	0.64*	0.62*	0.53*	0.60*
14 yrs	230	0.70*	0.69*	0.66*	0.58*	0.61*	0.60*
15 yrs	221	0.63*	0.65*	0.58*	0.56*	0.55*	0.49*
16 yrs	170	0.61*	0.61*	0.57*	0.54*	0.47*	0.58*

Note: N = 2,556.

Intraclass Correlation Coefficients (ICC) are designated as ≤ 0.40 poor to fair agreement, 0.41–0.60 moderate agreement, 0.61–0.80 good agreement, and 0.81–1.00 excellent agreement.

*p < .001.

**Table 9:** Two-way mixed effect model (absolute agreement, single measure) Intraclass Correlations (ICC) between Child Self-Report and Parent Proxy-Report for PedsQL™ 4.0 Generic Core Scales by Age for Healthy Sample

**Age**	**n**	**Total Score**	**Physical Health**	**Psychosocial Health**	**Emotional Functioning**	**Social Functioning**	**School Functioning**
5 yrs	558	0.46*	0.25*	0.56*	0.68*	0.43*	0.46*
6 yrs	703	0.43*	0.24*	0.53*	0.67*	0.40*	0.44*
7 yrs	635	0.42*	0.21*	0.54*	0.66*	0.40*	0.43*
8 yrs	575	0.55*	0.40*	0.62*	0.72*	0.51*	0.46*
9 yrs	553	0.57*	0.37*	0.64*	0.70*	0.56*	0.55*
10 yrs	533	0.68*	0.50*	0.73*	0.80*	0.64*	0.61*
11 yrs	400	0.52*	0.36*	0.60*	0.69*	0.48*	0.52*
12 yrs	405	0.63*	0.44*	0.70*	0.74*	0.57*	0.61*
13 yrs	320	0.58*	0.42*	0.66*	0.68*	0.55*	0.54*
14 yrs	296	0.56*	0.43*	0.64*	0.74*	0.54*	0.59*
15 yrs	284	0.67*	0.45*	0.76*	0.79*	0.59*	0.73*
16 yrs	137	0.60*	0.42*	0.68*	0.73*	0.46*	0.68*

Note: N = 5,399.

Intraclass Correlation Coefficients (ICC) are designated as ≤ 0.40 poor to fair agreement, 0.41–0.60 moderate agreement, 0.61–0.80 good agreement, and 0.81–1.00 excellent agreement.

*p < .001.

## Discussion

The results demonstrate that children as young as the 5 year old age subgroup can reliably and validly self-report their HRQOL when given the opportunity to do so with an age-appropriate instrument. Thus, although most available HRQOL instruments only include child self-report for ages 8 and older [1,2], the present findings indicate that 5, 6, and 7 year olds can reliably and validly self-report their HRQOL, comparable to older children and adolescents. It should be noted that even though the 5–7 age subgroups had the lowest coefficient alpha reliability coefficients for the age subgroups tested, the PedsQL™ child self-report instrument response scale for ages 5–7 is reworded and simplified to a 3-point Likert response scale, rather than the 5-point Likert response scale used for ages 8–18. Previous research suggests that 3-point response scales attenuate the achievable reliability coefficients relative to 5-point response scales [52]. This may explain in part the somewhat lower reliability coefficients for the 5–7 age subgroups in comparison to the 8–18 age subgroups. Finally, the relatively large number of missing data for the School Functioning Scale for the 5–7 subgroups may have further attenuated the potentially achievable reliability coefficients for these age subgroups [44]. Although Cronbach alpha represents the lower bound of the reliability of a measurement instrument, and is a conservative estimate of actual reliability [53], scales that do not approach or meet the 0.70 standard should be used only for descriptive analyses.

Although parent/child agreement has not typically been reported for children younger than 7 years of age for HRQOL instruments [31], the trend towards higher intercorrelations with increasing age in the present study is perhaps consonant with the trend towards higher scale reliabilities with increasing age for self-report. Lower scale reliabilities may attenuate the intercorrelations between self and proxy reporters. An additional possible explanation may be the greater verbal communication skills typically manifested with increasing developmental age. Further, while it might be expected that the intercorrelations between child and parent report across the physical, emotional, social and school functioning scales would follow the conceptualization that more observable domains (i.e., physical functioning) would yield higher agreement, this has not necessarily been the case in the published literature with other HRQOL instruments. In a comprehensive review, Eiser [54] found mixed results in terms of higher intercorrelations between self and proxy report of physical functioning across pediatric HRQOL instruments, with most studies demonstrating this effect, while some others did not.

Taken together, the evidence is quite compelling that parent proxy-report of child HRQOL, across the age subgroups reported herein, should be included to complement pediatric patient self-report as a secondary outcome measure, not to serve as a convenient substitute or proxy for pediatric patient PROs in pediatric clinical trials. Parent proxy-report should only be the primary outcome measure when the child is too young or ill or otherwise unable to self-report [36].

## Conclusion

Evidence now available on thousands of children demonstrates that pediatric patients as young as the 5 year old age subgroup can reliably and validly self-report their HRQOL when an age-appropriate measurement instrument is utilized. Pediatric patient-reported outcomes should be accepted as the standard for HRQOL measurement in pediatric clinical trials in which patient health-related quality of life is investigated. In this way, the voices of the children will be heard in matters pertaining to their health and well-being given the perspective that "some treatment effects are known only to the patient" [7]. Measuring perceived health from the perspective of children provides a level of accountability consistent with the Institute of Medicine report on the quality of care [55]. As the consumers of pediatric healthcare, children are uniquely positioned to give their perspectives on healthcare quality through their perceptions of their health-related quality of life.

## Abbreviations

HRQOL Health-Related Quality of Life

PedsQL™ Pediatric Quality of Life Inventory™

PRO Patient-Reported Outcomes

FDA Food and Drug Administration

## Competing interests

Dr. Varni holds the copyright and the trademark for the PedsQL™ and receives financial compensation from the Mapi Research Trust, which is a nonprofit research institute that charges distribution fees to for-profit companies that use the Pediatric Quality of Life Inventory™. The PedsQL™ is available at the PedsQL™ Website [56].

## Authors' contributions

JWV conceptualized the rationale and design of the study. JWV and CAL drafted the manuscript. CAL performed the statistical analyses. TMB participated in the statistical analyses. All authors read and approved the final manuscript.
